# The Impact of *FTO* Genetic Variants on Obesity and Its Metabolic Consequences is Dependent on Daily Macronutrient Intake

**DOI:** 10.3390/nu12113255

**Published:** 2020-10-23

**Authors:** Przemyslaw Czajkowski, Edyta Adamska-Patruno, Witold Bauer, Joanna Fiedorczuk, Urszula Krasowska, Monika Moroz, Maria Gorska, Adam Kretowski

**Affiliations:** 1 Clinical Research Centre, Medical University of Bialystok, Jana Kilinskiego 1, 15-089 Bialystok, Poland; przemyslaw.czajkowski@umb.edu.pl (P.C.); witold.bauer@umb.edu.pl (W.B.); urszula.krasowska@umb.edu.pl (U.K.); adamkretowski@wp.pl (A.K.); 2 Clinical Research Centre, Medical University of Bialystok Clinical Hospital, Marii Sklodowskiej-Curie 24A, 15-276 Bialystok, Poland; j.fiedorczuk@wp.pl (J.F.); monika_bakun@wp.pl (M.M.); 3 Department of Endocrinology, Diabetology and Internal Medicine, Medical University of Bialystok, Jana Kilinskiego 1, 15-089 Bialystok, Poland; mgorska25@wp.pl

**Keywords:** *FTO* gene, obesity, dietary protein, dietary carbohydrates, dietary fat, macronutrients, gene-diet interaction, glucose homeostasis

## Abstract

Numerous studies have identified the various fat mass and obesity-associated (*FTO*) genetic variants associated with obesity and its metabolic consequences; however, the impact of dietary factors on these associations remains unclear. The aim of this study was to evaluate the association between *FTO* single nucleotide polymorphisms (SNPs), daily macronutrient intake, and obesity and its metabolic consequences. From 1549 Caucasian subjects of Polish origin, genotyped for the *FTO* SNPs (rs3751812, rs8044769, rs8050136, and rs9939609), 819 subjects were selected for gene–diet interaction analysis. Anthropometric measurements were performed and total body fat content and distribution, blood glucose and insulin concentration during oral glucose tolerance test (OGTT), and lipid profile were determined. Macronutrient intake was analyzed based on three-day food records, and daily physical activity levels were evaluated using the International Physical Activity Questionnaire Long Form (IPAQ-LF). Our study shows that carriers of the GG genotype of rs3751812 presented lower body weight, body mass index (BMI), total body fat content, and hip and waist circumference and presented lower obesity-related markers if more than 48% of daily energy intake was derived from carbohydrates and lower subcutaneous and visceral fat content when energy intake derived from dietary fat did not exceed 30%. Similar results were observed for rs8050136 CC genotype carriers. We did not notice any significant differences in obesity markers between genotypes of rs8044769, but we did observe a significant impact of diet-gene associations. Body weight and BMI were significantly higher in TT and CT genotype carriers if daily energy intake derived from carbohydrates was less than 48%. Moreover, in TT genotype carriers, we observed higher blood glucose concentration while fasting and during the OGTT test if more than 18% of total energy intake was derived from proteins. In conclusion, our results indicate that daily macronutrient intake may modulate the impact of *FTO* genetic SNPs on obesity and obesity-related metabolic consequences.

## 1. Introduction

Obesity is a major public health problem worldwide [1] and a leading risk factor for type 2 diabetes mellitus in adolescents [2,3] and children [3,4]. It has already been established that the predictions for diabetes prevalence are not optimistic [5]. Moreover, the increasing prevalence of obesity is associated with lipid metabolism disturbances, such as high concentrations of total cholesterol and low-density lipoprotein (LDL) and low concentrations of high-density lipoprotein (HDL) [6]. Considering the above, obesity also increases the risk of cardiovascular disease [7].

In general, obesity is a result of imbalanced energy homeostasis, but genome-wide association studies have identified many single nucleotide polymorphisms (SNPs) in the fat mass and obesity-associated (*FTO*) gene, melanocortin-4 receptor (*MC4R*) gene, and other genes [8,9] that are associated with the risk of developing obesity. Among these genes, *FTO* has been reported as the gene with the strongest significant correlation with obesity [10]. The *FTO* gene is profoundly expressed in the hypothalamus region, which is involved in appetite regulation [11]. The associations between *FTO* genetic variants, dietary factors, and body weight gain are still under investigation, although it has been postulated that some *FTO* genetic variants may influence the risk of weight gain through larger amounts of consumed food [12] or appetite and satiety regulation [13]. *FTO* rs9939609 SNPs have been associated with increased macronutrient consumption, especially fat and carbohydrates, as well as total energy intake [11,14,15], but these genetic variants do not seem to influence energy expenditure [11,16]. Moreover, environmental factors such as diet may influence the associations between genetic risk and obesity development. Over the last decade, the study of dietary patterns and their relation to genetic risk of obesity has received more attention [17,18]; nevertheless, the associations between *FTO* single nucleotide polymorphisms and dietary patterns need further investigation [19]. Therefore, the aim of our study was to evaluate whether daily macronutrient intake could modify the association between genetic variations of the *FTO* gene and obesity and obesity-related metabolic consequences among the Polish population.

## 2. Materials and Methods

### 2.1. Participants

The study was conducted among 1549 Caucasian volunteers of Polish origin (18–79 years old) enrolled in the 1000PLUS Cohort Study (registered at www.clinicaltrials.gov as NCT03792685) from 2007 to 2019, described previously [20,21,22], which were seeking personalized nutrition for prevention of obesity and treatment of type 2 diabetes mellitus. Individuals who used to take medicines (weight loss, anti-diabetic, lipid-lowering, or any other medication that could have an impact on body weight, body fat content, blood glucose, and other investigated parameters) or diet supplements, which could affect the results, were not enrolled in this study. Subjects who reported endocrine, gastrointestinal, hepatic, renal, metabolic, immunological, or psychiatric disorders or who had bariatric surgery, which could have an impact on investigated parameters, were excluded from the study analysis as well. We excluded all subjects who used to take anti-diabetic (56 subjects, 6.8%) or lipid-lowering medications (47 subjects, 5.7%) and 109 (13.3%) with a previous history of prediabetes or diabetes, as potential cofounders, and others who met the exclusion criteria mentioned above. Moreover, individuals who followed any special diet or dietary pattern (vegetarian, vegan, Atkins, etc.) were not included in the analysis.

### 2.2. Anthropometric and Body Composition Measurements

The following anthropometric data were collected: body weight, height, and waist and hip circumference. Body mass index (BMI) was calculated using the following formula: body weight (kg) divided by height squared (m). Waist–hip ratio (WHR) was estimated by dividing waist circumference by hip circumference. Total body composition (including fat mass, fat-free mass, and skeletal muscle mass) was evaluated by the bioelectrical impedance method (InBody 220, Biospace, Korea). Body fat distribution analysis, including visceral adipose tissue (VAT) and subcutaneous adipose tissue (SAT) content, was performed by the multi-frequency bioimpedance method (Maltron BioScan 920-2, Maltron International Ltd., United Kingdom). The VAT/SAT ratio was calculated by dividing visceral adipose tissue by subcutaneous adipose tissue content. 

### 2.3. Blood Collection, Biochemical Analysis, and Calculations

Oral glucose tolerance tests (OGTTs) were performed in non-diabetic participants according to the World Health Organization (WHO) recommendations with a dose of 75 g oral glucose. The subjects were instructed to fast for 8–12 h prior to the test and to not restrict carbohydrate intake in the 3 days before the test. Blood was collected at 0, 30, 60, and 120 min after glucose administration. Blood samples were obtained and collected to evaluate the concentrations of plasma glucose, insulin, LDL, HDL, total cholesterol and triglyceride (TG), and hemoglobin A1c (HbA1c). The samples were prepared for assessment according to the laboratory kit instructions. Serum insulin concentrations were evaluated by immunoradiometric assay (INS-Irma, DIASource S.A., Belgium; Wallac Wizard 1470 Automatic Gamma Counter, PerkinElmer Life Sciences, Turku, Finland). Plasma glucose concentration was measured using the hexokinase enzymatic method (Cobas c111, Roche Diagnostics Ltd., Switzerland), and lipid profile was evaluated by enzymatic colorimetric assay using commercially available kits (Cobas c111, Roche Diagnostic Ltd., Switzerland). HbA1c levels were measured using high-performance liquid chromatography (HPLC) (D-10 Hemoglobin Testing System, Bio-Rad Laboratories Inc., Hercules, CA, USA; Bio-Rad, Marnes-la-Coquette, France).

The homeostatic model assessment of insulin resistance (HOMA-IR) was calculated following the standard formula: (fasting plasma glucose concentration (mmol/L)) × (fasting insulin concentration (μU/mL))/22.5.

### 2.4. Daily Physical Activity and Dietary Intake Analyses

To evaluate daily physical activity, the International Physical Activity Questionnaire-Long Form (IPAQ-LF) was used. Metabolic equivalent (MET, min per week) was determined using the following formula: (MET level) × (minutes of activity) × (events per week) [23]. Individuals were stratified as having a low, moderate, or high level of physical activity.

Subjects were asked to record 3-day food intake diaries and were instructed on how to estimate portion sizes of foods based on the provided color photograph albums of portion sizes. Moreover, the subjects were instructed on how to weigh the food, if possible. Daily carbohydrate, protein, fat, and total energy intake were estimated using Dieta 6.0 software (National Food and Nutrition Institute, Warsaw, Poland). Dieta software was developed and is continuously updated by the National Food and Nutrition Institute (Warsaw, Poland), and it is used to calculate the nutritional value of food and diets based on tables of the nutritional value of local food products and dishes. In order to study the interactions between genetic factors and diet, study participants were divided into 2 quantiles based on average daily protein, fat, and carbohydrate intake: lower and higher than median dietary protein intake (≤18% and >18% of total energy intake, respectively), lower and higher than median dietary carbohydrate intake (≤48% and >48% of total energy intake, respectively), and lower and higher than median dietary fat intake (≤30% and >30% of total energy intake, respectively).

### 2.5. Genetic Analyses

We genotyped 4 common *FTO* SNPs in rs3751812 (G > T), rs8044769 (C > T), rs8050136 (A > C), and rs9939609 (T > A). DNA was extracted from peripheral blood leukocytes using a classical salting-out method. The SNPs were genotyped with TaqMan SNP technology from a ready-to-use human assay library (Applied Biosystems, MA, USA) using a high-throughput genotyping system, OpenArray (Life Technologies, CA, USA). SNP analysis was performed in duplicate, following the manufacturer’s instructions. We used a sample without template as a negative control to detect possible false positive signals caused by contamination.

### 2.6. Ethics Statement

The study methods were carried out in accordance with the ethical standards on human experimentation and with the Helsinki Declaration of 1975 as revised in 1983. Written informed consent was obtained from all participants before inclusion in the study. The study protocol was approved by the local Ethics Committee of the Medical University of Bialystok, Poland (R-I-002/35/2009).

### 2.7. Statistical Analysis

Numerical data were summarized with number of observations (N), arithmetic mean, and standard deviation (SD). For categorical data, number of observations and frequency (percentage) were presented. Study participants were divided into quantiles based on average daily protein, carbohydrate, and fat intake, with the thresholds set as the median value of each parameter. Risk genotypes of the 4 common *FTO* SNPs were predefined based on the literature and our previous findings. Because of the relatively small sample size, we did not include a comparison of the allelic and genotypic frequencies and odds ratio calculations in this study. Continuous parameters were tested for normality with Shapiro-Wilk’s test as well as visual inspection. Homogeneity of variance across groups was studied using Levene’s test. Nonparametric tests were used for response variables that failed the mentioned statistical tests. Differences between selected parameters and dietary groups were then compared using analysis of variance (ANOVA) or Kruskal-Wallis test for numerical variables, with either Tukey’s or Dunn’s post-hoc test with Holm *p*-value adjustment (in case multiple pairwise tests were performed, or when there were multiple grouping variables, as presented in tables and figures), and chi-squared test for categorical variables. In order to study the hypothesis that the relationship between *FTO* genotypes and continuous responses varies in average daily protein, fat, and carbohydrate intake groups, we added (dietary macronutrient quantile) x (genotype) interaction terms to the multivariate linear regression models. These models were adjusted for age, sex, BMI (when applicable), total average energy intake (kcal/day), and physical activity. The Huber-White robust standard errors (HC1) were calculated. Model fit was estimated using R-squared values plus adjusted R-squared values. Some of the models were optimized by a stepwise backward elimination based on the Akaike information criterion (AIC). The statistical significance level was set at <0.05 for all 2-sided tests and multivariate comparisons. All calculations were prepared in R (version 4.0.2) [24].

## 3. Results

Our analysis identified 411 participants (50.2%) as having prediabetes or diabetes, without any previously known history of glucose homeostasis disturbance. 

For the diet-gene interaction analysis, we included data from 819 subjects (Supplementary Figure S1). The general clinical characteristics of the studied population are presented in Table 1, and characteristics stratified by investigated genotypes are presented in Table 2, Table 3 and Table 4. No significant deviation from the Hardy-Weinberg equilibrium was observed for any of the investigated SNPs (*p* > 0.05). Among the investigated *FTO* SNPs, some of the loci were in very strong linkage disequilibrium (D’ = 1.0 for rs8050136 and rs9939609) [25], so we present results for rs8050136.

Based on the demographic, anthropometric, behavioral (food intake and physical activity), and laboratory data, we observed that GG genotype carriers of rs3751812 (Table 2) and CC genotype carriers of rs8050136 (Table 3) presented significantly lower hip circumference. We also found that carriers of the TT rs8044769 genotype had the highest total cholesterol levels, and CT carriers presented the lowest percentage of daily energy intake from fat (Table 4).

We did not observe any other significant differences between studied genotypes; however, we noticed a tendency toward higher BMI, total body fat content, and waist circumference in TT genotype carriers of rs3751812 (Table 2) and AA genotype carriers of rs8050136 (Table 3). Between carriers of investigated genetic variants in rs8044769, we noted a tendency for differences in low-density lipoprotein cholesterol (LDL-cholesterol) concentration and daily physical activity level (Table 4).

### 3.1. Dietary Assessment

The 3-day food diaries were available from 662 subjects from the general cohort group and from 490 subjects who were genotyped for the investigated *FTO* SNPs. We did not find any differences between genotypes and dietary habits, except for the rs8044769 SNP. The heterozygous CT genotype carriers presented the lowest percentage of energy intake provided from dietary fat (Table 4).

We analyzed the interactions between dietary macronutrient intake and individual genotypes and their effect on continuous responses using multivariable linear regression models with the (dietary macronutrient quantile) × (genotype) interaction term. We observed that the association between selected genotypes and variables describing body composition (weight, BMI, and free fat mass) and the patient’s glycemic status (fasting glucose levels) varied in different dietary groups, confirming the hypothesis that the effects of diet and genotypes interact. The differences in median values of the selected responses and the interquartile ranges (IQRs) in different genotypic and dietary strata are presented using boxplots in the figures. These results were significant after adjustment for age, sex, BMI (where applicable), and total energy intake.

### 3.2. Association of rs3751812 Genetic Variants with Obesity, Anthropometric Measures, Lipid Profile, and Dietary Intake

**Figure 1:**
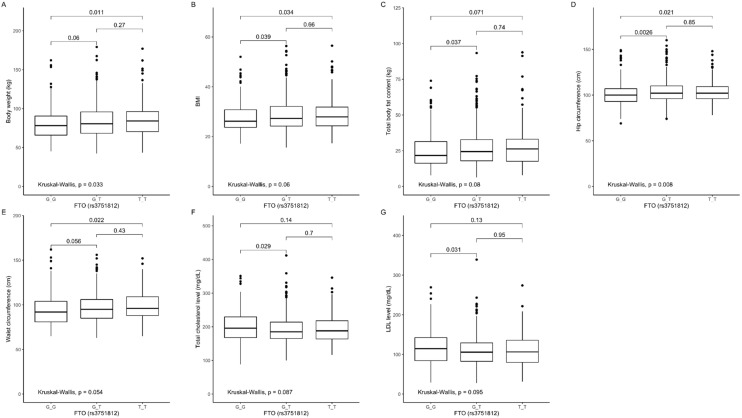
Association of fat mass and obesity-associated (*FTO*) genotype rs3751812 with (**A**) body weight (kg), (**B**) BMI (kg/m^2^), (**C**) total body fat content (kg), (**D**) hip circumference (cm^3^), (**E**) waist circumference (cm^3^), (**F**) total cholesterol level (mg/dL), (**G**) LDL level (mg/dL).

The comparison between genotypes showed that carriers of the GG genotype presented lower body weight (Figure 1A), BMI (Figure 1B), total body fat content (Figure 1C), and hip (Figure 1D) and waist (Figure 1E) circumference, but higher total cholesterol (Figure 1F) and LDL-cholesterol (Figure 1G) levels, when compared to the GT genotype carriers, and lower body weight (Figure 1A), BMI (Figure 1B), and hip (Figure 1D) and waist (Figure 1E) circumference when compared to the TT genotype carriers.

**Figure 2:**
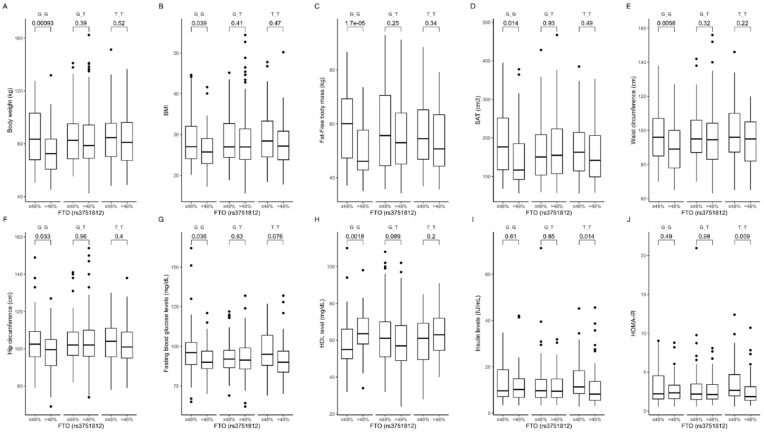
Association of *FTO* genotypes rs3751812 with (**A**) body weight (kg), (**B**) BMI (kg/m^2^), (**C**) fat-free body mass (kg), (**D**) subcutaneous adipose tissue (SAT) (cm^3^), (**E**) waist circumference (cm^3^), (**F**) hip circumference (cm^3^), (**G**) fasting blood glucose level (mg/dL), (**H**) HDL level (mg/dL), (**I**) fasting blood insulin level (IU/mL), and (**J**) HOMA-IR by dietary carbohydrate intake strata: ≤48% and >48% of total daily energy intake. HOMA-IR, Homeostatic Model Assessment for Insulin Resistance.

Based on the analysis of the interactions between rs3751812 genotypes and carbohydrate intake, we observed that GG genotype carriers presented lower body weight (Figure 2A), BMI (Figure 2B), fat-free mass levels (Figure 2C), subcutaneous fat content (Figure 2D), and waist (Figure 2E) and hip (Figure 2F) circumference, as well as lower fasting blood glucose (Figure 2G) and higher HDL-cholesterol (Figure 2H) levels, when they were stratified to the group with higher than median carbohydrate intake. Moreover, we noted that TT carriers in the group with higher than median carbohydrate intake presented lower fasting insulin levels (Figure 2I) and HOMA-IR values (Figure 2J) compared to participants who were stratified to the group with lower than median carbohydrate intake. The interaction effect of (carbohydrate diet group) × (rs3751812 genotype) on body composition, anthropometric measures, and lipid profile was statistically significant with *p*-value < 0.05.

**Figure 3:**
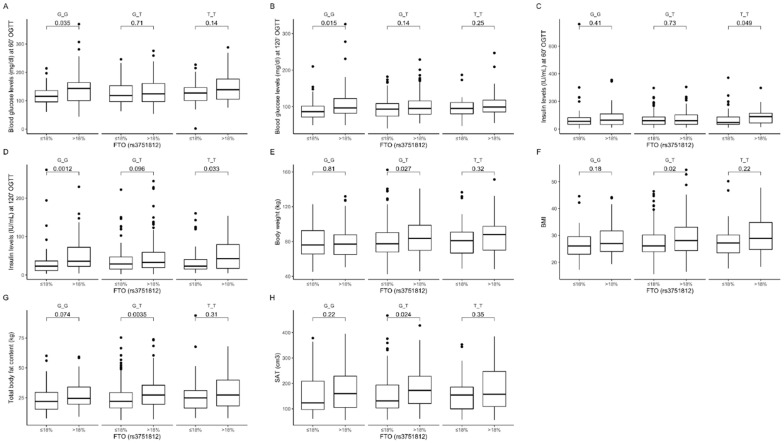
Association of dietary protein intake ≤18% and >18% of total daily energy intake with blood glucose level (mg/dL) at (**A**) 60 min and (**B**) 120 min of OGTT; insulin level (IU/mL) at (**C**) 60 min and (**D**) 120 min of OGTT; (**E**) body weight (kg); (**F**) BMI (kg/m^2^); (**G**) total body fat content (kg); and (**H**) SAT (cm^3^) in *FTO* rs3751812 genotype carriers.

Our further analysis showed that GG carriers in the group with lower than median protein intake presented lower blood glucose levels at 60 (Figure 3A) and 120 min (Figure 3B) of OGTT, while TT genotype participants in the group with higher than median protein intake presented higher insulin levels at 60 min (Figure 3C). We also observed higher insulin levels at 120 min of OGTT in GG and TT genotype carriers stratified to the group with higher than median protein intake (Figure 3D). The heterozygous GT genotype carriers in the group with lower than median dietary protein intake presented lower body weight (Figure 3E), BMI (Figure 3F) and total body (Figure 3G) and subcutaneous (Figure 3H) fat content. Using linear modeling, we found a significant interaction effect of (protein diet group) × (rs3751812 genotype) on body composition (*p*-value < 0.05) and blood glucose and insulin levels (*p*-value < 0.01) at 60 and 120 min of OGTT.

**Figure 4:**
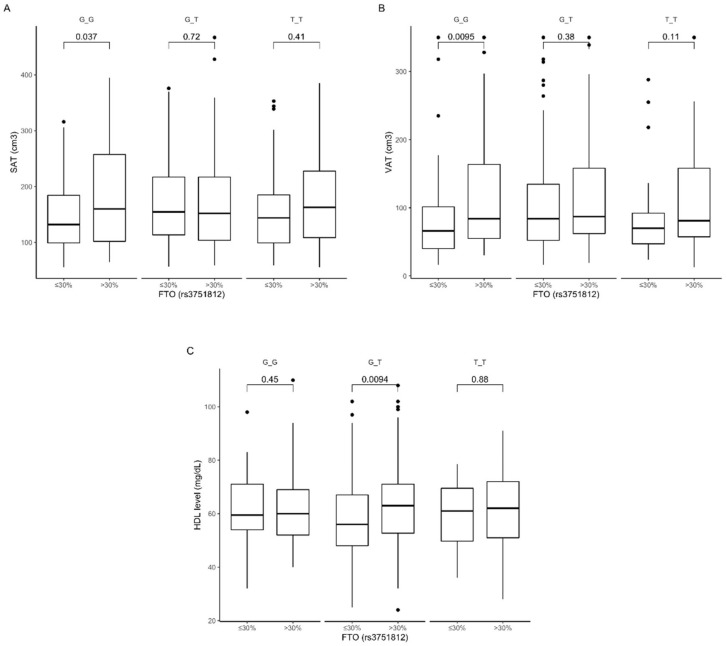
Association of dietary fat intake ≤30% and >30% of total daily energy intake with (**A**) SAT (cm^3^), (**B**) VAT (cm^3^), and (**C**) HDL level (mg/dL) in *FTO* rs3751812 genotype carriers.

Analyzing the dietary fat intake, we noted that carriers of the GG genotype stratified to the group with lower than median fat intake presented lower subcutaneous (Figure 4A) and visceral (Figure 4B) fat content. Surprisingly, we observed that GT genotype carriers showed higher HDL levels (Figure 4C) when they were stratified to the group with higher than median fat intake. We did not observe any other association with dietary fat intake. The interaction effect of (fat diet group) × (rs3751812 genotype) on subcutaneous and visceral fat content was statistically significant, with *p*-value < 0.01, as well as on HDL levels (*p*-value < 0.002).

### 3.3. Association of rs8050136 Genetic Variants with Obesity, Anthropometric Measures, Lipid Profile, and Dietary Intake

**Figure 5:**
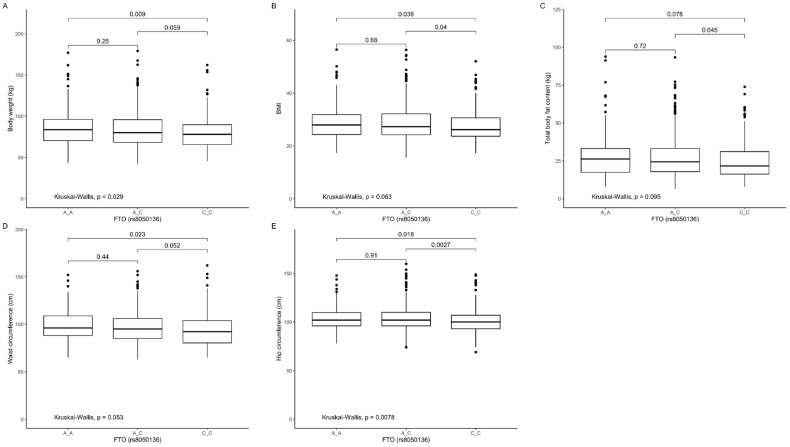
Association of *FTO* genotypes rs8050136 with (**A**) body weight (kg), (**B**) BMI (kg/m^2^), (**C**) total body fat content (kg), (**D**) waist circumference (cm^3^), (**E**) hip circumference (cm^3^).

Our analysis showed that CC genotype carriers presented significantly lower body weight (Figure 5A), BMI (Figure 5B), and waist (Figure 5D) and hip (Figure 5E) circumference compared to TT, and significantly lower BMI (Figure 5B), total body fat content (Figure 5C), and hip (Figure 5E) circumference when compared to CT genotype carriers.

**Figure 6:**
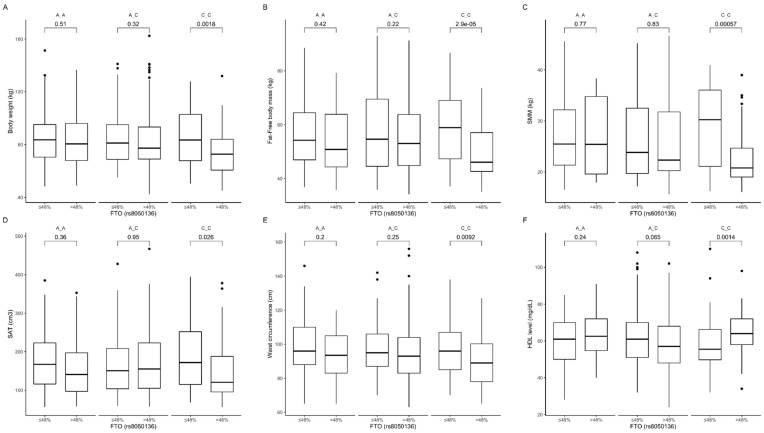
Association of *FTO* genotypes rs8050136 with (**A**) body weight (kg), (**B**) fat-free body mass (kg), (**C**) skeletal muscle mass (SMM) (kg), (**D**) SAT (cm^3^), (**E**) waist circumference (cm^3^), and (**F**) HDL level (mg/dL) by dietary carbohydrate intake strata: ≤48% and >48% of total daily energy intake.

Based on the analysis of the interactions between rs8050136 genotypes and carbohydrate intake, we observed that CC genotype carriers in the group with higher than median carbohydrate intake presented lower body weight (Figure 6A), fat-free body mass level (Figure 6B), skeletal muscle mass content (Figure 6C), subcutaneous fat content (Figure 6D), and waist circumference (Figure 6E) and higher HDL-cholesterol level (Figure 6F). The interaction effect of (carbohydrate diet group) × (rs8050136 genotypes) on body composition (*p*-value < 0.05) and HDL (*p*-value < 0.01) was statistically significant.

**Figure 7:**
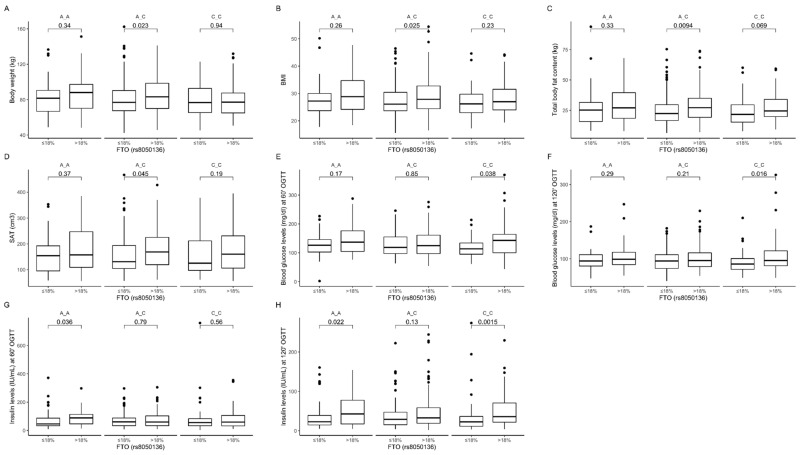
Association of dietary protein intake ≤18% and >18% of total daily energy intake with (**A**) body weight (kg); (**B**) BMI (kg/m^2^); (**C**) total body fat content (kg); (**D**) SAT (cm^3^); blood glucose level (mg/dL) at (**E**) 60 min and (**F**) 120 min of OGTT; and insulin level (IU/mL) at (**G**) 60 min and (**H**) 120 min of OGTT in *FTO* rs8050136 genotype carriers.

We observed that AC genotype carriers stratified to the group with lower than median protein intake presented lower body weight (Figure 7A), BMI (Figure 7B), total body fat content (Figure 7C), and subcutaneous fat content (Figure 7D). Additionally, we noticed that CC genotype carriers who were stratified to the group with higher than median protein intake presented higher blood glucose levels at 60 min (Figure 7E) and 120 min (Figure 7F) of OGTT. Higher insulin levels were observed in AA genotype carriers at 60 min (Figure 7G) and 120 min (Figure 7H) of OGTT, and in CC genotype carriers at 120 min (Figure 7H) of OGTT. The interaction effect of (protein diet group) × (rs8050136 genotypes) on body composition, anthropometric measures, and lipid profile was statistically significant with *p*-value < 0.05.

**Figure 8:**
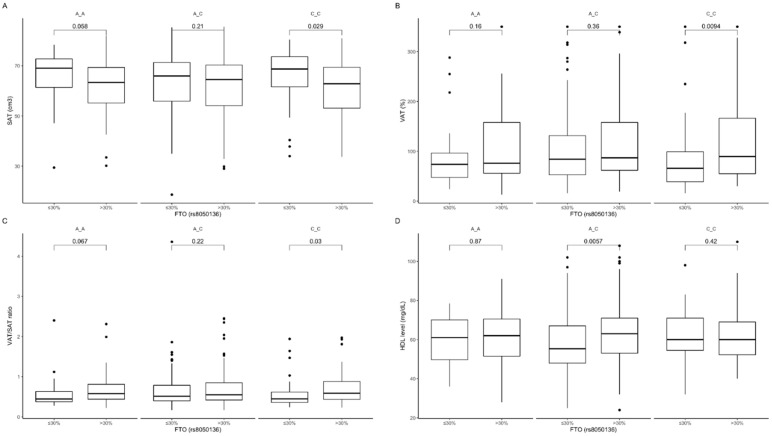
Association of dietary fat intake ≤30% and >30% of total daily energy intake with (**A**) SAT (cm^3^), (**B**) VAT (%), (**C**) SAT/VAT ratio, and (**D**) HDL level (mg/dL) in *FTO* rs8050136 genotype carriers.

The analysis of dietary fat intake showed that carriers of the CC genotype stratified to the group with lower than median fat intake presented surprisingly higher subcutaneous fat content (Figure 8A) and lower visceral fat content (Figure 8B), as well as lower VAT/SAT ratio (Figure 8C). In AA genotype carriers, we noticed similar tendencies (Figure 8A,C). In carriers of the AC genotype stratified to the group with higher than median fat intake, we observed higher HDL-cholesterol levels (Figure 8D) compared to those who were stratified to the group with lower than median fat intake. The interaction effect of (fat diet group) × (rs3751812 genotype) on subcutaneous and visceral fat content as well as it ratio was statistically significant with *p*-value < 0.01, the interaction effect on HDL was the largest, with the *p*-value of 0.001.

### 3.4. Association of rs8044769 Genetic Variants with Obesity, Anthropometric Measures, Lipid Profile, and Dietary Intake

**Figure 9:**
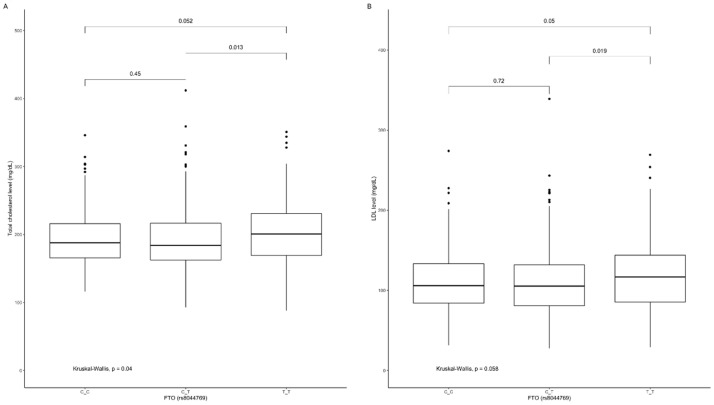
Association of *FTO* genotypes rs8044769 with (**A**) total cholesterol level (mg/dL) and (**B**) LDL level (mg/dL).

The TT genotype carriers of rs8044769 presented significantly higher total cholesterol (Figure 9A) and LDL-cholesterol (Figure 9B) levels when compared to CT genotype carriers, and similar marginally significant results were noted between TT and CT genotype carriers (Figure 9A,B). 

**Figure 10:**
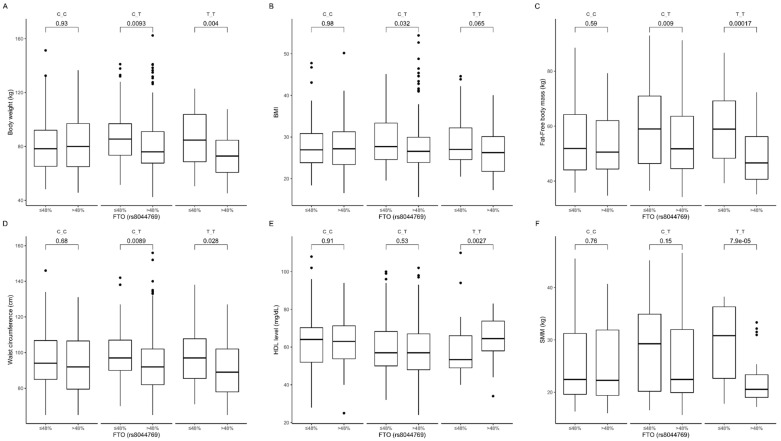
Association of *FTO* genotypes rs8044769 with (**A**) body weight (kg), (**B**) BMI (kg/m^2^), (**C**) fat-free body mass (kg), (**D**) waist circumference (cm^3^), (**E**) HDL level (mg/dL), and (**F**) SMM (kg) by dietary carbohydrate intake strata: ≤48% and >48% of total daily energy intake.

The gene-diet interaction analysis showed that TT and CT genotype carriers presented higher body weight (Figure 10A), BMI (Figure 10B), fat-free body mass (Figure 10C), and waist circumference (Figure 10D) when they were stratified to the group with lower than median carbohydrate intake. Homozygous TT carriers in the group with lower than median carbohydrate intake also presented lower HDL-cholesterol levels (Figure 10E) and surprisingly higher skeletal muscle mass content (Figure 10F). We did not notice any association between percentage of daily energy intake provided from carbohydrates and investigated metabolic parameters in CC genotype carriers. The interaction effect of (carbohydrate diet group) × (rs8044769 genotype) on body composition, anthropometric measures, and HDL was statistically significant with *p*-value < 0.05.

**Figure 11:**
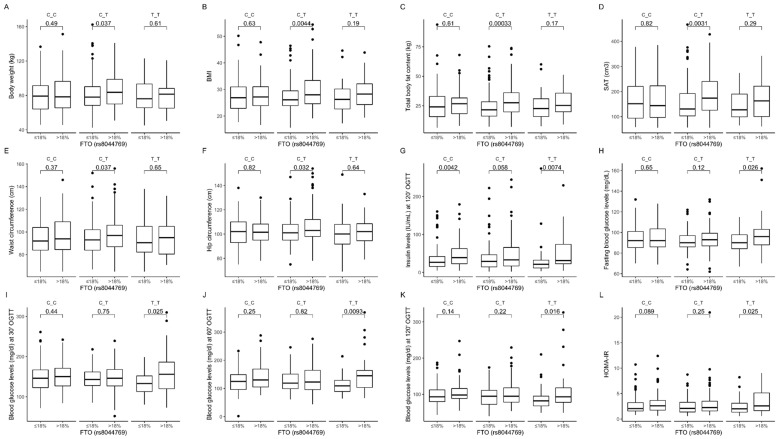
Association of dietary protein intake ≤18% and >18% of total daily energy intake with (**A**) body weight (kg); (**B**) BMI (kg/m^2^); (**C**) total body fat content (kg); (**D**) SAT (cm^3^); (**E**) waist circumference (cm^3^); (**F**) hip circumference (cm^3^); (**G**) insulin level (IU/mL) at 120 min of OGTT; (**H**) fasting blood glucose level (mg/dL); blood glucose level (mg/dL) at (**I**) 30 min, (**J**) 60 min, and (**K**) 120 min of OGTT; and (**L**) HOMA-IR in *FTO* rs8044769 genotype carriers.

Among individuals in the group with higher than median protein intake, we observed that heterozygous CT carriers showed higher body weight (Figure 11A), BMI (Figure 11B), total body fat content (Figure 11C), subcutaneous fat content (Figure 11D), waist circumference (Figure 11E), and hip circumference (Figure 11F). In addition, we noted that both CC and TT homozygous carriers presented higher insulin concentration at 120 min (Figure 11G); however, higher fasting blood glucose levels (Figure 11H) and blood glucose levels at 30 min (Figure 11I), 60 min (Figure 11J), and 120 min (Figure 11K) of OGTT were noted only in TT genotype carriers stratified to the group with higher than median protein intake. We noted significantly higher values of HOMA-IR in TT genotype carriers, and the same tendency in CC genotype carriers, when dietary protein provided >18% of total energy compared to subjects who were stratified to the group with lower than median protein intake (Figure 11L). The interaction effect of (protein diet group) × (rs8044769 genotype) on body composition, and blood glucose and insulin levels was statistically significant with *p*-value < 0.05.

**Figure 12:**
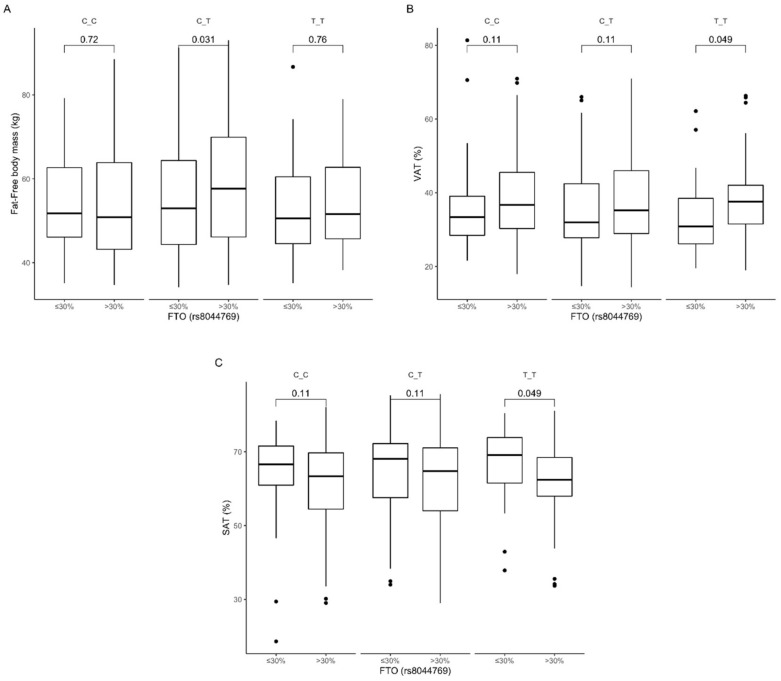
Association of dietary fat intake ≤30% and >30% of total daily energy intake with (**A**) fat-free body mass (kg), (**B**) SAT (cm^3^), and (**C**) VAT (%) in *FTO* rs8044769 genotypes carriers.

An association with dietary fat intake was observed in carriers of the heterozygous CT genotype stratified to the group with higher than median fat intake, who presented higher fat-free body mass content (Figure 12A). TT genotype carriers had higher visceral fat content (Figure 12B) and lower subcutaneous fat content (Figure 12C) when they were stratified to the group with higher than median dietary fat intake compared to carriers of the same genotype in the group with lower than median dietary fat intake. The interaction effect of (fat diet group) × (rs8044769 genotype) on subcutaneous and visceral fat content was statistically significant with *p*-value < 0.05.

## 4. Discussion

Over the past few decades, public awareness in the field of nutrition and physical activity has increased, but obesity and its comorbidities are still serious international health problems [26]. It is widely known that the *FTO* gene is an established genetic susceptibility locus for the risk of obesity development [27]. However, the association between the *FTO* gene and dietary factors is still unclear and there is a scientific need to investigate the associations between environmental and genetic risk factors and their interactions and roles in obesity development and treatment. In our study, we demonstrated an interplay between *FTO* genetic variants and dietary carbohydrate, protein, and fat intake, and the impact of these interactions on body weight, body fat content and distribution, and other anthropometric measures, as well as on glucose homeostasis and lipid profile, in a Polish population of adults. For our study, we chose some of the most common SNPs based on previously published results [25,26,27]. 

We observed a protective effect of the GG genotype of rs3751812 against obesity, but GG genotype carriers presented higher total cholesterol and LDL-cholesterol levels. It was shown previously that *FTO* rs3751812 risk allele T is related to increased BMI and body fat distribution compared to the protective allele G [28]. However, our results indicate that carrying the GG genotype leads to more beneficial effects if more than 48% of total diet energy comes from carbohydrates; then, we could observe significantly lower obesity-related parameters. We did not notice any difference in total body fat content, and lower body weight and BMI could be associated with noted lower fat-free mass. Nevertheless, we did not observe any adverse effects of lower fat-free mass content, since we also noted lower fasting glucose and higher HDL-cholesterol concentration. The impact of the TT genotype, which appears to be a risk genotype for obesity, seems to not be related to carbohydrate intake, except for the associations with fasting insulin concentration and HOMA-IR level.

We also noted associations between dietary protein intake, SNPs, and metabolic parameters for all investigated genetic variants of rs3751812, indicating that we can observe more beneficial results if dietary protein provides no more than 18% of total daily energy intake. These observations are worth underlining, especially in light of the current interest in high-protein diets. Based on the results that we noted for high-risk TT genotype carriers (rs3751812) in the group with higher than median protein intake, including higher post-absorptive insulin levels and higher fasting insulin concentrations and HOMA-IR levels in subjects in the group with lower than median carbohydrate intake, we can hypothesize that these individuals should avoid high-protein, low-carbohydrate diets. It is also worth noting that for TT genotype subjects, we did not observe any differences or adverse effects that would depend on dietary fat intake. Moreover, it has already been found that carriers of minor allele T rs3751812 present a lower risk of obesity when they adhere better to a Mediterranean diet, which consists of higher daily consumption of fats, especially from olive oil and nuts [29]. We noted an association with dietary fat intake only in GG and GT genotypes carriers for body fat distribution and HDL-cholesterol level. In these subjects, if daily energy intake derived from fat was more than 30%, we could observe higher visceral and subcutaneous fat content.

Our study also shows significant associations of *FTO* SNPs in rs8050136 with the investigated markers of obesity. The CC genotype has been shown to play a protective role, and CC genotype carriers presented significantly lower body weight, BMI, total body fat content, and waist and hip circumference. Our observations are in line with results from previous studies [30,31,32,33]. Although numerous studies have shown an association of SNPs in rs8050136 with higher values of BMI and waist and hip circumference, the impact of dietary intake on this association is still unclear. In our study, we did not notice any crucial differences in daily dietary macronutrient intake between genotypes. Nevertheless, we observed that CC genotype subjects were more susceptible to the beneficial effects when carbohydrate in their diets provided more than 48% of total daily energy intake and no more than 18% came from dietary protein. Moreover, dietary fat should be limited to less than 30% of total daily energy intake to avoid visceral fat accumulation in these subjects. We also observed that AC genotype carriers of rs8050136 stratified to the group with lower than median dietary fat intake presented lower HDL-cholesterol levels. Bego et al. [34] observed that the risk A allele of rs8050136 was significantly associated with decreased HDL-cholesterol levels in control subjects in type 2 diabetes studies. We did not notice differences between studied genotypes, but surprisingly, we observed lower HDL-cholesterol concentrations in AC genotype carriers only when they followed a diet with less than 30% of energy from dietary fat. In these subjects, when dietary fat provided more than 30% of total energy, then HDL concentrations were significantly higher, without any differences in total cholesterol or LDL-cholesterol levels. We did not evaluate the source of fat, if the diet was rich in saturated or unsaturated fatty acids, or what could explain our observations, because it is well known that various types of fatty acids have different impacts on plasma lipid concentrations [35].

We observed that dietary protein intake might have an impact on obesity-related parameters only in heterozygous CA carriers of rs8050136, while in AA and CC genotype carriers, on glucose homeostasis-related markers, and in all cases, beneficial effects were noted when dietary protein provided no more than 18% of total daily energy intake. It has already been reported by Park et al. that the association of the rs8050136 risk variant may be partially mediated by macronutrient intake [36], but only the association with percentage of energy derived from fat has been detected.

The analysis of differences between genetic variants of rs8044769 showed only that TT genotype carriers presented higher total and LDL-cholesterol levels. We did not notice any other differences between genotypes; however, TT and CT genotype carriers presented lower body weight, BMI, and waist circumference and higher HDL-cholesterol levels when more than 48% of total daily energy was derived from carbohydrates, even if fat-free mass and skeletal muscle mass were also significantly lower. The obesity-related parameters seemed to be associated with dietary protein intake only in heterozygous CT carriers and in TT genotype carriers with glucose homeostasis-related parameters. All of the noted associations were more beneficial if daily energy derived from dietary protein intake did not exceed 18%. Moreover, our study suggests that CT and TT genotype carriers should also not consume dietary fat exceeding 30% of total daily energy intake, to avoid visceral fat accumulation. We did not observe any significant associations for CC genotype carriers that could depend on dietary protein or fat intake, except insulin levels at 120 min of OGTT, which were significantly higher in subjects stratified to the group with higher than median protein intake. There is a very limited number of available studies on *FTO* rs8044769 genetic variants, and some of them present these variants as BMI-associated SNPs [37,38], but in others, the authors did not observe such a relationship [39]. Moreover, considering the fact that all associations can vary with ethnicity, gender, dietary intake, and some other factors, studies in larger and more diverse populations are needed.

The present study has several strengths. As far as we know, this is one of the first studies to present interactions between *FTO* SNPs rs3751812, rs8044769, rs8050136, and rs9939609 and macronutrient intake, and the effect of these relationships on obesity and obesity-related complications. Another strength of our study is that it is based on a relatively large population. It is also worth noting that it has been shown that *FTO* genetic variants may influence dietary factors [14,40,41,42] or dietary fat intake [36,43,44], while other studies did not confirm these associations [12,15]. In general, we did not observe any significant differences in macronutrient intake between studied genotypes, which can also be interpreted as a strength of our study, because we can exclude the possibility that our results might be affected by the impact of different macronutrient intake on gene expression and the activation of different metabolic pathways. Nevertheless, several limitations of our study also need to be addressed. Some parts of our results are based on self-reported data, such as three-day diaries of food intake, and it has been shown that obese people tend to underreport or misreport their total dietary intake, especially fatty foods and foods rich in carbohydrates [45]. However, dietary questionnaires are the only known implements available for large-scale population investigations so far. Moreover, only Caucasian individuals were recruited for our study; therefore, in order to verify our findings in other ethnic groups, the data should be replicated in other populations.

Our results, if confirmed in larger populations of different ethnic groups, may have also practical clinical implications. Based on our observations, we can recommend that carriers of GG genotype of rs3751812 and CC genotype of rs8050136 follow diets in which no less than 48% of daily energy intake is derived from carbohydrates and no more than 30% from dietary fat. Moreover, carriers of TT and CT genotypes of rs8044769 should avoid diets in which carbohydrates provide less than 48% of total energy, whereas carriers of TT genotype should avoid diets in which proteins provide more than 18% of total daily energy, to prevent glucose homeostasis disturbances. These recommendations seem to be highly important, since we noticed that the mean amounts of macronutrients in the diets of the investigated population were mostly less than 48% of total energy intake for carbohydrates, more than 30% for dietary fat, and more than 18% for proteins, which may have adverse effects for carriers of the above-mentioned genotypes.

## 5. Conclusions

In conclusion, our findings provide new insights into the role of the interactions between diet and *FTO* SNPs in the risk of obesity and its metabolic consequences. Advances in this field bring us closer to the development of genome-customized diet recommendations to prevent obesity. Detecting *FTO* risk genotype carriers and modifying dietary intake according to the genetic profile may be a novel, efficient strategy to prevent obesity development.

## Figures and Tables

**Table 1:** Study group characteristics.

Study Group Characteristics	
N	819
Age	42.1 (14.5)
Female/male (%)	52.5/47.5
BMI (kg/m^2^)	28.5 (6.6)
<25.0	273 (33.9%)
25.0–29.9	278 (34.5%)
≥30.0	255 (31.6%)
Total body fat content (kg)	27.1 (13.8)
Total body fat content (%)	31.4 (9.6)
Waist circumference (cm)	96.2 (17.2)
Hip circumference (cm)	103.3 (12.7)
WHR	0.928 (0.088)
Visceral fat (cm^3^)	108.4 (80.6)
Visceral fat (%)	37.1 (12.1)
Subcutaneous fat (cm^3^)	167.9 (81.7)
Subcutaneous fat (%)	62.8 (12.3)
Visceral/subcutaneous fat ratio	0.669 (0.443)
Total cholesterol	195.4 (46.1)
HDL	59.7 (14.9)
LDL	112.0 (40.0)
TG	118.8 (95.1)
Fasting blood glucose level (mg/dL)	98.8 (23.9)
History of prediabetes or diabetes	
Yes	411 (50.2%)
No	408 (49.8%)
Dietary assessment (n)	490
Daily energy intake (kcal)	1792.5 (697.4)
Daily energy from protein (%)	18.9 (4.8)
Daily energy from fat (%)	31.2 (7.5)
Daily energy from carbohydrates (%)	47.6 (8.6)
Daily physical activity level	
Low	60 (7.3%)
Moderate	173 (21.1%)
High	586 (71.6%)

Data presented as mean and standard deviation (SD). BMI, body mass index; HDL, high-density lipoprotein; LDL, low-density lipoprotein; TG, triglycerides; WHR, waist–hip ratio.

**Table 2:** Characteristics of participants stratified by rs3751812 genotypes.

rs3751812	G/G	G/T	T/T	*p*-Value *
N	211	420	181	
Genotype frequency	0.26	0.52	0.22	>0.05
Age	40.5 (14.2)	41.2 (14.7)	39.5 (14.3)	0.33
Female (%)	53.8% (0.49)	53.8% (0.50)	47.8% (0.50)	0.36
BMI (kg/m^2^)	27.6 (6.0)	28.7 (6.8)	28.9 (6.8)	0.060
<25.0	81 (38.8%)	136 (32.9%)	53 (29.9%)	0.410
25.0–29.9	69 (33.0%)	141 (34.1%)	66 (37.3%)	
≥30.0	59 (28.2%)	136 (32.9%)	58 (32.8%)	
Total body fat content (kg)	25.3 (12.4)	27.6 (13.8)	28.2 (15.2)	0.080
Total body fat content (%)	30.6 (9.1)	31.8 (9.6)	31.6 (10.3)	0.377
Waist circumference (cm)	94.3 (17.5)	96.7 (17.2)	97.5 (16.7)	0.054
Hip circumference (cm)	101.3 (12.4)	104.2 (13.0)	103.8 (12.5)	0.008
WHR	0.927 (0.091)	0.925 (0.088)	0.937 (0.085)	0.327
Visceral fat (cm^3^)	103.0 (81.0)	110.0 (79.9)	112.3 (83.0)	0.379
Visceral fat (%)	36.4 (11.8)	37.5 (12.4)	37.2 (11.7)	0.587
Subcutaneous fat (cm^3^)	163.5 (83.1)	167.2 (80.5)	175.0 (82.7)	0.401
Subcutaneous fat (%)	63.7 (11.7)	62.3 (12.9)	62.8 (11.7)	0.557
Visceral/subcutaneous fat ratio	0.642 (0.406)	0.687 (0.475)	0.665 (0.413)	0.554
Total cholesterol	202.7 (56.0)	191.7 (41.3)	194.0 (43.2)	0.070
HDL	60.7 (14.1)	59.8 (15.6)	59.5 (14.5)	0.662
LDL	117.3 (43.3)	109.4 (37.8)	111.2 (41.8)	0.095
TG	123.8 (143.9)	111.9 (69.7)	116.3 (61.9)	0.289
Blood glucose level during OGTT (mg/dL)				
0 min	96.8 (24.1)	95.6 (18.3)	97.1 (20.8)	0.914
30 min	147.0 (44.3)	145.0 (31.6)	150.1 (35.6)	0.312
60 min	132.3 (56.0)	129.5 (46.3)	134.2 (46.3)	0.380
120 min	100.7 (46.1)	99.1 (32.1)	98.8 (31.0)	0.621
History of prediabetes or diabetes				
Yes	103 (48.8%)	209 (49.8%)	95 (52.5%)	0.751
No	108 (51.2%)	211 (50.2%)	86 (47.5%)	
Dietary assessment (n)	126	259	101	
Daily energy intake (kcal)	1807.2 (732.3)	1766.9 (676.0)	1837.4 (713.4)	0.849
Daily energy from protein (%)	18.7 (4.4)	19.0 (4.9)	19.1 (4.9)	0.901
Daily energy from fat (%)	31.2 (7.2)	30.9 (7.5)	31.9 (7.8)	0.568
Daily energy from carbohydrates (%)	47.6 (7.7)	47.8 (9.1)	46.8 (8.5)	0.662
Daily physical activity level				
Low	16 (7.6%)	25 (6.0%)	18 (9.9%)	0.302
Moderate	50 (23.7%)	83 (19.8%)	40 (22.1%)	
High	145 (68.7%)	312 (74.3%)	123 (68.0%)	

Data presented as mean and standard deviation (SD), number of observations, and frequency. BMI, body mass index; HDL, high-density lipoprotein; LDL, low-density lipoprotein; OGTT, oral glucose tolerance test; TG, triglycerides; WHR, waist-hip ratio. * Holm-adjusted Kruskal-Wallis/ANOVA *p*-values.

**Table 3:** Characteristics of participants stratified by rs8050136 genotypes.

rs8050136	C/C	A/C	A/A	*p*-Value *
N	209	424	182	
Genotype frequency	0.26	0.52	0.22	>0.05
Age	40.2 (14.1)	41.3 (14.8)	39.4 (14.3)	0.24
Females (%)	54.3% (0.49)	53.4% (0.49)	47.7% (0.50)	0.36
BMI (kg/m^2^)	27.6 (6.1)	28.7 (6.8)	28.9 (6.8)	0.063
<25.0	80 (38.6%)	138 (33.2%)	54 (30.2%)	0.422
25.0–29.9	69 (33.3%)	140 (33.7%)	67 (37.4%)	
≥30.0	58 (28.0%)	138 (33.2%)	58 (32.4%)	
Total body fat content (kg)	25.3 (12.4)	27.5 (13.8)	28.2 (15.2)	0.095
Total body fat content (%)	30.6 (9.1)	31.8 (9.6)	31.6 (10.4)	0.465
Waist circumference (cm)	94.2 (17.6)	96.7 (17.2)	97.4 (16.6)	0.053
Hip circumference (cm)	101.2 (12.5)	104.1 (13.0)	103.8 (12.4)	0.008
WHR	0.927 (0.092)	0.925 (0.088)	0.936 (0.085)	0.382
Visceral fat (cm^3^)	103.3 (81.5)	110.2 (80.0)	111.8 (82.5)	0.381
Visceral fat (%)	36.4 (11.7)	37.6 (12.5)	37.1 (11.6)	0.570
Subcutaneous fat (cm^3^)	163.7 (83.5)	166.9 (80.8)	175.3 (83.1)	0.405
Subcutaneous fat (%)	63.7 (11.6)	62.2 (13.0)	62.9 (11.6)	0.540
Visceral/subcutaneous fat ratio	0.641 (0.404)	0.690 (0.477)	0.662 (0.410)	0.536
Total cholesterol	201.9 (56.1)	192.1 (41.4)	193.7 (43.1)	0.153
HDL	60.8 (14.0)	59.6 (15.7)	59.7 (14.4)	0.422
LDL	116.3 (43.3)	109.9 (37.9)	111.1 (41.8)	0.189
TG	124.1 (144.3)	113.2 (71.1)	115.1 (61.3)	0.491
Blood glucose level during OGTT (mg/dL)				
0 min	96.8 (24.2)	95.8 (18.5)	96.8 (20.4)	0.922
30 min	146.6 (44.5)	145.2 (31.7)	150.1 (35.4)	0.263
60 min	131.7 (56.4)	129.9 (46.1)	133.9 (46.1)	0.413
120 min	100.2 (46.3)	99.3 (32.2)	98.6 (30.9)	0.493
History of prediabetes or diabetes				
Yes	100 (47.8%)	213 (50.2%)	96 (52.7%)	0.628
No	109 (52.2%)	211 (49.8%)	86 (47.3%)	
Dietary assessment (n)	103	264	123	
Daily energy intake (kcal)	1820.5 (734.9)	1759.6 (673.3)	1853.9 (716.3)	0.645
Daily energy from protein (%)	18.7 (4.5)	19.0 (4.9)	19.0 (4.9)	0.855
Daily energy from fat (%)	31.2 (7.2)	30.9 (7.5)	31.9 (7.8)	0.506
Daily energy from carbohydrates (%)	47.6 (7.7)	47.9 (9.1)	46.8 (8.4)	0.572
Daily physical activity level				
Low	16 (7.7%)	25 (5.9%)	19 (10.4%)	0.179
Moderate	50 (23.9%)	83 (19.6%)	40 (22.0%)	
High	143 (68.4%)	316 (74.5%)	123 (67.6%)	

Data presented as mean and standard deviation (SD), number of observations, and frequency. BMI, body mass index; HDL, high-density lipoprotein; LDL, low-density lipoprotein; OGTT, oral glucose tolerance test; TG, triglycerides; WHR, waist-hip ratio. * Holm-adjusted Kruskal-Wallis/ANOVA *p*-values.

**Table 4:** Characteristics of participants stratified by rs8044769 genotypes.

rs8044769	C/C	C/T	T/T	*p*-Value *
N	270	406	138	
Genotype frequency	0.33	0.50	0.17	>0.05
Age	40.4 (14.8)	41.2 (14.6)	39.6 (13.7)	0.54
Females (%)	51.8% (0.50)	51.9% (0.50)	56.2% (0.49)	0.65
BMI (kg/m^2^)	28.5 (6.8)	28.7 (6.7)	27.8 (6.1)	0.534
<25.0	88 (33.2%)	131 (32.8%)	51 (37.5%)	0.862
25.0–29.9 (kg/m^2^)	92 (34.7%)	143 (35.8%)	43 (31.6%)	
≥30.0 (kg/m^2^)	85 (32.1%)	126 (31.5%)	42 (30.9%)	
Total body fat content (kg)	27.7 (15.0)	27.2 (13.6)	25.8 (11.9)	0.676
Total body fat content (%)	31.7 (10.1)	31.4 (9.6)	31.1 (9.0)	0.893
Waist circumference (cm)	96.2 (17.2)	96.8 (17.3)	94.6 (16.8)	0.429
Hip circumference (cm)	103.3 (12.6)	104.0 (13.0)	101.5 (12.2)	0.189
WHR	0.928 (0.089)	0.927 (0.088)	0.929 (0.089)	0.980
Visceral fat (cm^3^)	109.2 (78.3)	110.3 (83.7)	99.8 (72.5)	0.648
Visceral fat (%)	37.7 (11.8)	36.9 (12.3)	36.4 (12.0)	0.617
Subcutaneous fat (cm^3^)	168.6 (83.3)	169.7 (82.4)	160.9 (74.2)	0.773
Subcutaneous fat (%)	62.3 (11.8)	63.0 (12.8)	63.7 (11.8)	0.598
Visceral/subcutaneous fat ratio	0.689 (0.492)	0.662 (0.421)	0.641 (0.408)	0.590
Total cholesterol	193.8 (40.1)	191.7 (42.7)	206.7 (62.7)	0.029
LDL	111.0 (38.6)	109.6 (38.7)	119.8 (46.5)	0.058
HDL	60.7 (14.7)	59.2 (15.5)	60.4 (13.8)	0.176
TG	110.5 (58.7)	114.6 (73.4)	132.6 (170.6)	0.689
Blood glucose level during OGTT (mg/dL)				
0 min	96.6 (20.0)	96.1 (21.0)	96.0 (20.1)	0.910
30 min	149.6 (35.1)	143.7 (32.0)	148.8 (47.8)	0.229
60 min	133.3 (45.4)	128.7 (47.3)	133.5 (59.3)	0.303
120 min	98.6 (31.8)	99.3 (30.7)	100.6 (53.0)	0.147
History of prediabetes or diabetes				
Yes	143 (53.0%)	201 (49.5%)	64 (46.4%)	0.420
No	127 (47.0%)	205 (50.5%)	74 (53.6%)	
Dietary assessment (n)	157	248	83	
Daily energy intake (kcal)	1775.7 (646.2)	1792.7 (735.8)	1816.0 (686.3)	0.791
% of daily energy from protein	18.9 (4.8)	19.2 (5.0)	18.4 (4.1)	0.608
% of daily energy from fat	32.5 (7.3)	30.1 (7.5)	31.9 (7.4)	0.005
% of daily energy from carbohydrates	46.5 (8.5)	48.3 (8.9)	47.1 (8.1)	0.164
Daily physical activity level				
Low	23 (8.5%)	26 (6.4%)	10 (7.2%)	0.060
Moderate	55 (20.4%)	77 (19.0%)	41 (29.7%)	
High	192 (71.1%)	303 (74.6%)	87 (63.0%)	

Data presented as mean and standard deviation (SD), number of observations, and frequency. BMI, body mass index; HDL, high-density lipoprotein; LDL, low-density lipoprotein; OGTT, oral glucose tolerance test; TG, triglycerides; WHR, waist-hip ratio. * Holm-adjusted Kruskal-Wallis/ANOVA *p*-values.

## Supplementary Materials

The following are available online at https://www.mdpi.com/2072-6643/12/11/3255/s1, Figure S1: Study flowchart diagram.
