# Supplementary figures and images for: The Impact of FTO Genetic Variants on Obesity and Its Metabolic Consequences is Dependent on Daily Macronutrient Intake
Source: Nutrients. 2020 Oct 23;12(11):3255. doi: 10.3390/nu12113255 (PMC7690875; doi:10.3390/nu12113255)

**Online Supporting Material.**

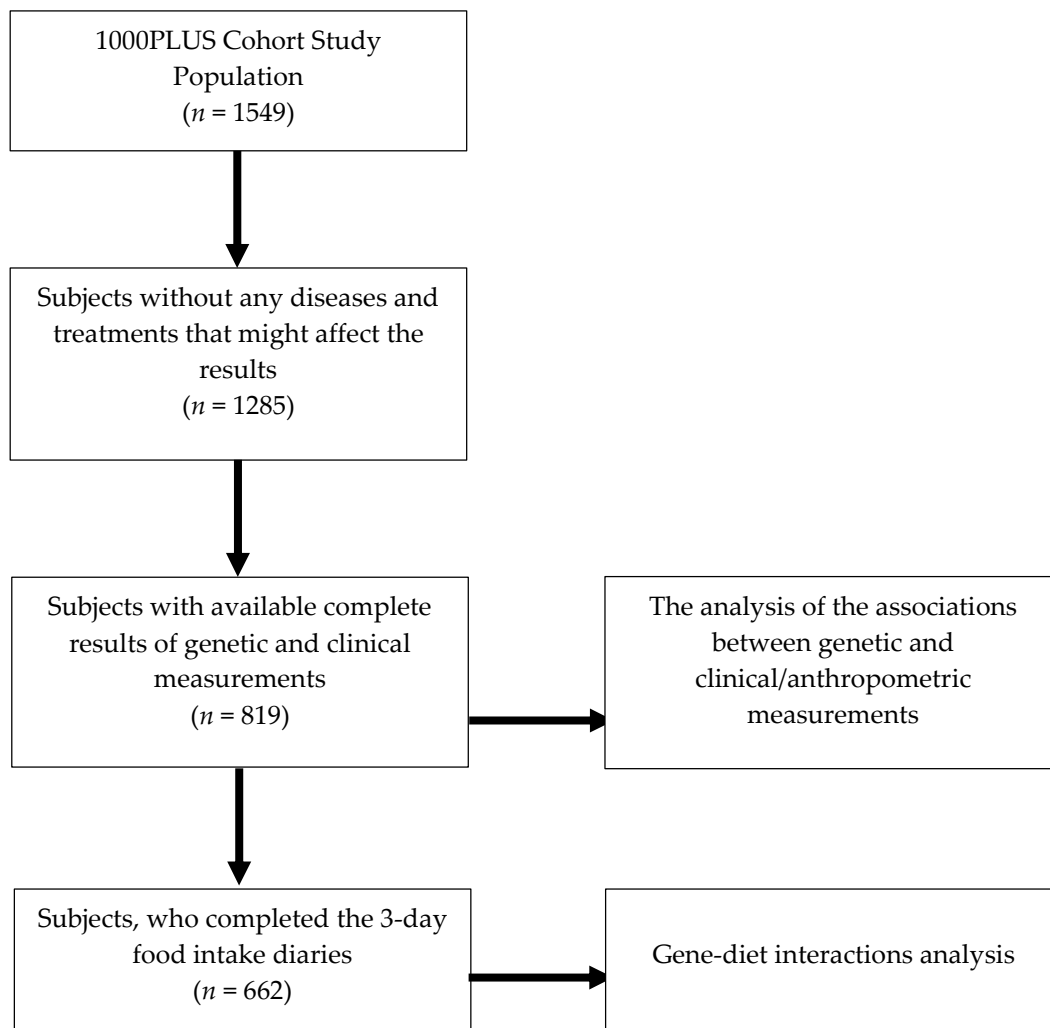

**Figure 1.** Study flowchart diagram.

Supplement: Supplementary file 1 [file nutrients-12-03255-s001.pdf]
